# Using Social Media to Engage Knowledge Users in Health Research Priority Setting: Scoping Review

**DOI:** 10.2196/29821

**Published:** 2022-02-21

**Authors:** Surabhi Sivaratnam, Kyobin Hwang, Alyssandra Chee-A-Tow, Lily Ren, Geoffrey Fang, Lindsay Jibb

**Affiliations:** 1 Michael G Degroote School of Medicine McMaster University Hamilton, ON Canada; 2 Child Health Evaluative Sciences Hospital for Sick Children Toronto, ON Canada; 3 Faculty of Health Sciences McMaster University Hamilton, ON Canada; 4 Faculty of Dentistry University of Toronto Toronto, ON Canada; 5 Lane Medical Library Stanford University Stanford, CA United States; 6 Faculty of Applied Science and Engineering University of Toronto Toronto, ON Canada; 7 Lawrence S Bloomberg Faculty of Nursing University of Toronto Toronto, ON Canada

**Keywords:** social media, research priority-setting, knowledge user, scoping review

## Abstract

**Background:**

The need to include individuals with lived experience (ie, patients, family members, caregivers, researchers, and clinicians) in health research priority setting is becoming increasingly recognized. Social media–based methods represent a means to elicit and prioritize the research interests of such individuals, but there remains sparse methodological guidance on how best to conduct these social media efforts and assess their effectiveness.

**Objective:**

This review aims to identify social media strategies that enhance participation in priority-setting research, collate metrics assessing the effectiveness of social media campaigns, and summarize the benefits and limitations of social media–based research approaches, as well as recommendations for prospective campaigns.

**Methods:**

We searched PubMed, Embase, Cochrane Library, Scopus, and Web of Science from database inception until September 2021. Two reviewers independently screened all titles and abstracts, as well as full texts for studies that implemented and evaluated social media strategies aimed at engaging knowledge users in research priority setting. We subsequently conducted a thematic analysis to aggregate study data by related codes and themes.

**Results:**

A total of 23 papers reporting on 22 unique studies were included. These studies used Facebook, Twitter, Reddit, websites, video-calling platforms, emails, blogs, e-newsletters, and web-based forums to engage with health research stakeholders. Priority-setting engagement strategies included paid platform–based advertisements, email-embedded survey links, and question-and-answer forums. Dissemination techniques for priority-setting surveys included snowball sampling and the circulation of participation opportunities via internal members’ and external organizations’ social media platforms. Social media campaign effectiveness was directly assessed as number of clicks and impressions on posts, frequency of viewed posts, volume of comments and replies, number of times individuals searched for a campaign page, and number of times a hashtag was used. Campaign effectiveness was indirectly assessed as numbers of priority-setting survey responses and visits to external survey administration sites. Recommendations to enhance engagement included the use of social media group moderators, opportunities for peer-to-peer interaction, and the establishment of a consistent tone and brand.

**Conclusions:**

Social media may increase the speed and reach of priority-setting participation opportunities leading to the development of research agendas informed by patients, family caregivers, clinicians, and researchers. Perceived limitations of the approach include underrepresentation of certain demographic groups and addressing such limitations will enhance the inclusion of diverse research priority opinions in future research agendas.

## Introduction

### Background

The need to meaningfully engage individuals with lived experience (ie, patients, family members, caregivers, clinicians, researchers, and other advocates; henceforth referred to as knowledge users) in the conduct of health research—defined as research that includes clinical and basic medical sciences, such as care-based research, systems research, and preventative research—is being increasingly recognized by the scientific community. In particular, it is recognized that these individuals should be included at the onset of the research process, with the aim of developing research that meets the needs of individuals with lived experiences [1]. In fact, the lack of involvement of these individuals in such research priority setting has been identified as a key contributor to difficulties in effectively translating research findings into clinical practice and policy [2].

In parallel, the use of social media—defined as any web-based platform or mobile app through which users can engage with others—is gaining considerable traction within the research community, as researchers increasingly access Facebook, Twitter, and YouTube to support participant recruitment and other research activities [3]. The benefits of research-related social media use include enhanced connectivity between researchers and participants and the potential for rapid diffusion of scientific knowledge to target audiences [4]. The nature of web-based survey methods may also enhance anonymity for participants within the research process, potentially promoting the collection of more valid data [5]. Particularly, data collected via the web may be less vulnerable to contextual biases that can arise in focus group settings or when researchers administer surveys in-person [5].

In light of such potential benefits, a growing body of literature describing the use of social media to elicit and prioritize research uncertainties from knowledge users is emerging [6]. However, there remains sparse methodological guidance on how best to conduct social media efforts and their corresponding effectiveness in developing knowledge user–built research agendas [7].

### Objective and Research Questions

Through this knowledge user–driven scoping review, we aim to identify studies that implemented and evaluated social media campaigns that promote participation in setting priorities for health research to address three overarching research questions:

1. What social media–based strategies have been used to enhance knowledge user participation in health research priority setting?
2. What metrics (direct and indirect) have been used to assess the effectiveness of these social media campaigns in securing knowledge user participation?
3. From the perspectives of those conducting social media–based research priority-setting campaigns, what are the benefits and limitations of the method, as well as recommendations for future campaigns?

## Methods

### Overview

An internal protocol was developed for this review. Our reporting process was conducted in accordance with the PRISMA-ScR (Preferred Reporting Items for Systematic Reviews and Meta-Analyses extension for scoping reviews) guidelines [8].

### Search Strategy and Selection of Studies

A comprehensive search strategy was developed in consultation with a tertiary hospital librarian (LR). We conducted tailored searches in PubMed, Embase, Cochrane Library, Scopus, and Web of Science. We searched all databases from their inception to September 14, 2021. Multimedia Appendix 1 shows the search strategy. Intradatabase and interdatabase duplicates were removed electronically. Using Covidence (Veritas Health Innovation), titles and abstracts were screened independently by 2 trained authors (KH and SS) according to our eligibility criteria. In cases of conflicting opinions on eligibility, studies were moved to full-text screening. Full-text articles were then screened independently by 2 authors (KH and SS). Any eligibility disagreements were resolved by consensus through discussion by at least three authors (AC, KH, SS, and LJ). The reference lists of relevant studies were also scanned to find other applicable papers.

### Selection Criteria

We included studies (1) that discussed strategies to promote social media–based health research priority setting among key stakeholders and knowledge users and (2) measured the effectiveness of such strategies directly or indirectly. There were no restrictions on the language, country, and year of publication, nor the research content focus, as priority-setting research is cross-disciplinary. Although no explicit restrictions were placed on the language, the included studies were dominated by English language–based social media campaigns. We defined social media as any web-based platform or mobile app through which users can interact and engage with others. We defined knowledge users as patients (or potential patients), caregivers, clinicians, and other advocates (eg, health researchers). We excluded (1) studies where the purpose of the social media campaign did not include knowledge user engagement (eg, social campaigns used to disseminate smoking cessation information to knowledge users) [9]; (2) studies where the research prioritization campaign did not involve social media (dissemination techniques solely involved telephone calls, flyer distribution, etc); and (3) abstracts, dissertations, protocols, systematic reviews, scoping reviews, or case studies.

### Data Extraction and Management

A standard electronic data collection form was created and piloted with our group, after which data extraction occurred independently (KH and SS). Discrepancies between the collected data were resolved through discussion with 3 authors (LJ, SS, and KH).

### Data Analyses

We used descriptive statistics to summarize quantitative study data and an inductive thematic analysis to synthesize qualitative data [10]. Our data collection form was uploaded to NVivo (version 12.6.0; QSR International) for analysis and was read through multiple times by 2 authors (KH and SS) who had previous experience with thematic analyses. One author (SS) then coded qualitative text within the table on a segment-by-segment basis. At frequent meetings, a second author (KH) reviewed the coding decisions using a constant comparative approach adapted from Thorne [11]. As a group, we (KH, SS, and LJ) then collapsed these codes into subthemes and themes based on the between-code relationships and in accordance with our research questions.

## Results

### Overview

**Figure 1:**
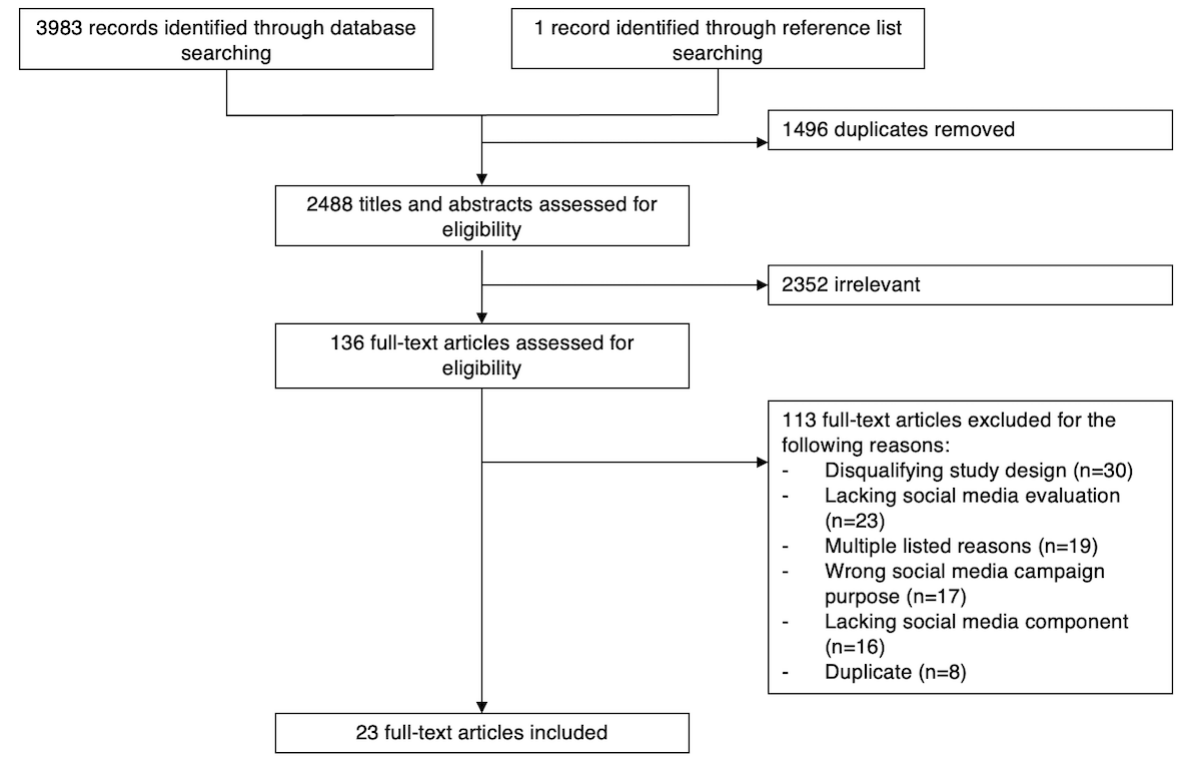
Study screening flowchart.

**Figure 2:**
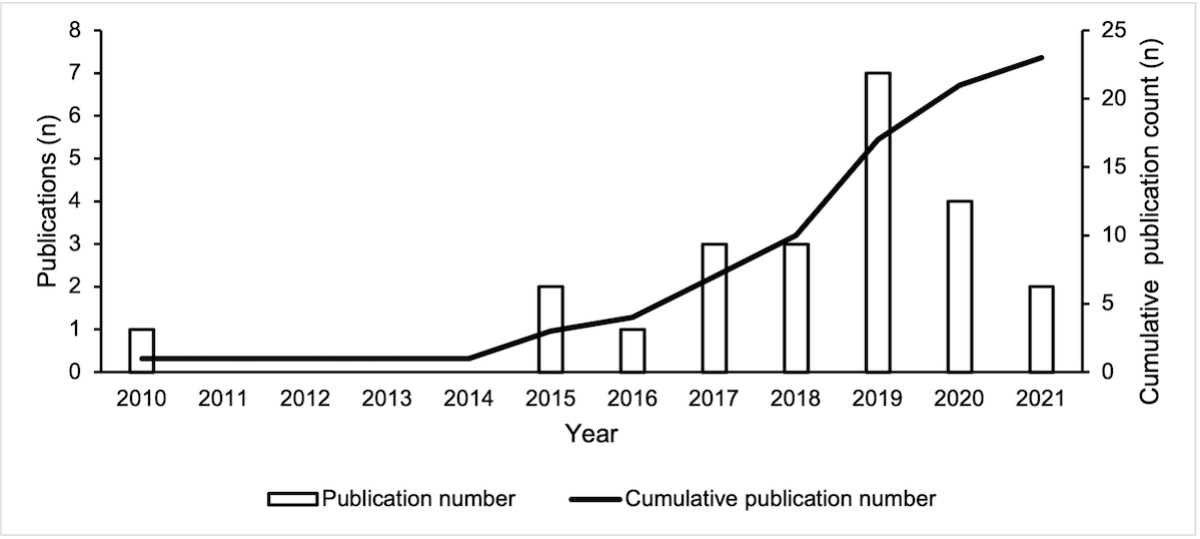
Social media–based research prioritization publication trend.

Figure 1 outlines our study identification process. Overall, 23 papers reporting on 22 unique studies were included in this review. The number of published studies increased steadily over time until 2020, which was the last complete publication year (Figure 2).

Included studies were conducted in 46 countries, most commonly in the United States (11/23, 48%), the United Kingdom (7/23, 30%), and Canada (5/23, 22%). Studies described participation by 13,640 individuals (median 332; range 31-4601), with sample size data missing from 4% (1/23) of the studies. Across studies, the median percentage of female participants was 77.28% (7404/9581). Sex data were missing from 52% (12/23) of the studies. Age data were variably reported and missing from 57% (13/23) of the studies; therefore, data were not collated. Sex data were missing from 39% (9/23) of the studies. Included studies used a variety of social media platforms to gather research priorities, including websites, emails, Facebook, Twitter, e-newsletters, web-based flyers, Survey Monkey, ExpertLens, blogs, YouTube, Choicebook, Instagram, WhatsApp, Snapchat, and web-based forums. The most common platforms used in the included studies were websites (12/23, 52%) and Facebook (9/23, 39%). The median length of a study’s social media campaign, when reported, was 3.5 months (range 1-24 months). Table 1 summarizes the characteristics of the included studies.

**Table 1:** Study characteristics (N=23).

Study	Year; country	Sample, N	Age and sex	Social media platform	Social media target group	Purpose for social media use	Duration of social media use	Social media outreach (eg, emails sent and posts made)	Social media analytics (outcomes)	Survey response rate	Outcomes of campaign in terms of research-priority gathering
Allsop et al [12]	2019; 32 countries within Africa	51	Not stated	Website and emails	Members of the African Palliative Care Association and individuals who work in palliative care	To identify (1) current mobile health use in palliative care, (2) potential barriers to use, and (3) priorities for research development	May to August 2016 (4 months)	101 organizations were emailed with web-based survey links	Not stated	51 (100%) survey responses (50.5% response rate)	Research priorities successfully identified
Correll et al [13]	2020; United States	365	Not stated	Website, emails, and other	Patients and caregivers of children (age ≥13 years)	To identify what research topics were most important to patients and caregivers of children with JM^a^, JA^b^, and cSLE^c^	November 2016, January 2017, and March 2017 for JM, AF^d^, and LFA^e^, respectively (5 months)	19,176 emails were sent	Not stated	441 survey responses	Research priorities successfully identified
Dyson et al [14]	2017; Canada and Portugal	110	Median age 35 years; 90% (99/110) women, 10% (11/110) men	Website, Facebook, and Twitter	Caregivers of children aged 0-17 years	To identify the outcome priorities of parents of children who had experienced an acute respiratory infection	December 2013 to March 2014 (4 months)	Creation of website, Facebook, and Twitter page or posts with embedded survey links	Website visits (5207); 3.9% view rate	110 (100%) survey respondents	Research priorities successfully identified
Dyson et al [15]	2017; Canada and Portugal	110	Median age 35 years; 90% (99/110) women, 10% (11/110) men	Website, Facebook, and Twitter	Caregivers of children aged 0-17 years	To identify the outcome priorities of parents of children who had experienced an acute respiratory infection	December 2013 to March 2014 (4 months)	Creation of website, Facebook, and Twitter page or posts with embedded survey links	Survey site visits (5027); Facebook page likes (104); and Twitter following (52 new followers)	110 (100%) survey respondents	Research priorities successfully identified
Eberman et al [16]	2019; United States	4601; 87 (1.89%) for focus groups, 4514 (98.11%) for survey	Age not stated; 55.05% (2533/4601) women, 43.40% (1997/4601) men, and 0.61% (28/4601) no indication	Newsletters via email	Athletic trainers	To identify research priorities and unify research with clinical practice to improve patient care and advance the profession	January 30, 2017 to March 16, 2017 (2 months)	48,752 emails were sent	Started the survey (5131, 10.5%); agreed to participate (4514, 9.3%); and completed the questionnaire (3910, 86.6%)	4514 (100%) research participants (9.3% response rate)	Research priorities successfully identified
Han et al [17]	2019; United States	332	Median age 51 years; 100% (332/332) women	Newsletters via web, website, Facebook, Twitter, web-based flyers, and emails	Females aged ≥18 years	To identify diabetes type 1 or 2 or prediabetes health research priorities	November 2016 to June 2017 (8 months)	904 website posts	Survey link clicks (421); comments on posts (904); total likes (530); total searches (167); and resource download (671)	332 (100%) research participants	Identified high priority research areas for women living with diabetes
Han et al [18]	2017; United States	332	Median age 49 years; 100% (332/332) women	Newsletters via web, website, Facebook, Twitter, web-based flyers, and emails	Females aged ≥18 years	To identify diabetes type 1 or 2 or prediabetes health research priorities	Not stated	551 emails were sent	Tag clicks (497); reposts and comments (872); voted for posts (540); searched for resources (167); and downloaded resources (671)	332 (100%) survey respondents (84% response rate)	The researchers identified 11 high priority categories of topics that were discussed on the DiabetesSistersVoices community
Healy et al [19]	2018; United Kingdom and Ireland	790	Age not stated; 71% (561/790) women, 28.98% (229/790) men	Website, emails, and Twitter	People invited to participate in a randomized trial or participated in Trial Steering Committees, front line randomized trials staff and investigators, and people familiar with trial methodology	To identify priority research questions related to trial recruitment	July 2016 to August 2016 (1 month)	Not stated	Not stated	790 (100%) respondents	List of top 10 trial recruitment uncertainties, determined by those directly involved in trials, were identified
Kim et al [20]	2018; United States	360	Age not stated; 60% (216/360) women, 40% (144/360) men	ExpertLens (ie, expert opinion forums), emails, and other	Patient, patient advocate, clinician, and researcher stakeholders	To determine engagement of stakeholders in research related to heart failure, obesity, and Kawasaki disease	18 months	Not stated	Not stated	84% response rate	Research priority successfully identified
Kriss et al [21]	2019; United States	207	Not stated	Email	Experts in global, regional, and national or subnational health	To identify research priorities for achieving disease elimination goals in the context of measles and rubella	October 17 to November 4, 2016 (approximately 1 month)	774 emails were sent	Not stated	207 (100%) respondents	Four main research priorities within the field of measles and rubella
Morris et al [22]	2015; United Kingdom	475	Not stated	Website, newsletters, and emails with embedded links	Children with neurodisability, caregivers, and clinicians	To identify and prioritize research questions regarding ways to improve the health and well-being of children and young people with neurodisability	Not stated	Creation of website and emails were sent with embedded links	Not stated	369 respondents (78% response rate)	Successfully established top 3 research priorities
Morse et al [23]	2021; United States	31	Mean age 15 years; 55% (17/31) women, 45% (14/31) men	Email and social media platforms (not specified)	Parents of children with medical complexity	To (1) ascertain parents’ perceived characteristics of child pain experiences, (2) determine the extent to which parents feel that caregivers adequately address pain, and (3) identify ways in which pain collaboration between parents and caregivers may be improved	August 2018 to February 2019 (6 months)	Posting institutional review board–approved message on primary investigator’s social media page	Social media post shares (n=30)	Not stated	Established research priorities
Normansell et al [5]	2015; United Kingdom	57	Not stated	Survey Monkey, Facebook, Twitter, website, and other	Patients, caregivers, and health care professionals with expertise in this discipline	To identify research priorities in asthma	August 6 to September 5, 2014 (1 month)	Not stated	“Obtained a large number of responses in a short timeperiod with potentially wide geographical reach”	Not stated	Developed a list of priority Cochrane Reviews
Oesophago-Gastric Anastomosis Study Group [24]	2020; United Kingdom	363	Not stated	WhatsApp and email	OGAA^f^ committee, national leaders, and engaged clinicians from high-, low-, and middle-income countries	To prioritize future research areas of unmet clinical need in RCTs^g^ to reduce anastomotic leaks	September to November 2019 (3 months)	Posted on organizations’ social media accounts	Not stated	Not stated	Established research priorities
Rowbotham et al [25]	2019; worldwide	482	Not stated	Twitter	Patients, their caregivers, and clinicians	To identify research priorities for cystic fibrosis	March 2016 to January 2017 (10 months)	320 tweets	Twitter followers gained (n=732); total number of views (n=151,000); engagements with hashtag (n=1806); and followers (n=1160)	Not stated	Top 10 list for research in CF^h^ was established
Russell et al [26]	2016; Canada	96	Not stated	Facebook	Family members of children	To exchange knowledge on project planning and research direction and translate research knowledge on disabilities and medical complexity	June 2014 to March 2015 (10 months)	432 Facebook posts were published	96 Facebook members; posts were generally seen by all group members; median likes (n=3); and comments (n=4)	49 respondents (51% response rate)	Provided researchers with an opportunity to consult families of children with special needs to receive guidance and hear issues that are important to them. Research priorities not identified
Salmi et al [27]	2020; United States	36	Not stated	Twitter, emails, blog posts, and Facebook groups	Patients with brain tumor and their care partners (ie, family members and friends who care for patients)	To describe the use of Twitter to complement in-person stakeholder engagement and report emerging themes from qualitative analysis of tweet chats on quality of life needs and palliative care opportunities for patients with brain tumor	April 2018 (1 month)	Two 60-minute scheduled live chat on Twitter	417 tweets by participants in first session and 355 tweets by participants in second session	N/A^i^	Research priorities, in the form of qualitative themes, were successfully identified
Shalhub et al [28]	2020; United States, United Kingdom, and Canada	300	Not stated	Blogs and website	Patients and their caregivers	To understand patient needs and determine the research methods best suited to study the adverse health implications associated with vascular Ehlers-Danlos syndrome	January 2018 and April 2018 (2 months)	Not stated	Facebook members in secret group (n=363) and Facebook followers (n=80,573)	Not stated	Optimal modality for research participation and methodologies for building trust in the research teams were identified
Shields et al [29]	2010; Canada	>800	Not stated	Choicebook, message board, blog, YouTube, Facebook, and email	Residents of and health service providers in northwestern Ontario	To engage the disperse population of northwestern Ontario in health care priority setting	Not stated	YouTube video welcome message; weekly blogs; and weekly participation update reports	“Hits” on website platform (n=2500); website views (n=2000); and >800 participants	Not stated	Findings identified new or additional research priorities for health network
Siefried et al [30]	2021; Australia	47	Mean age 42 years; 45% (21/47) women, 45% (21/47) men, and 5% (2/47) other or preferred not to say	Newsletter, emails with embedded links, Twitter, and website	Consumers, family, friends, caregivers, clinicians, researchers, policymakers, industry, research funders, institutions, organizations, law enforcement, border control, and other community members interested in the topic of methamphetamine	To identify clinical research priorities for methamphetamine and emerging drugs of concern in Australia, to guide the work of the National Centre for Clinical Research on Emerging Drugs	February 2019 to March 2019 (1 month)	Newsletter with embedded link were sent to mailing list and recipients of emails were invited to forward the email to other interested parties	Not stated	Not stated	Research themes and priorities were successfully identified
Sinclair et al [31]	2019; Croatia, France, Germany, Italy, the Netherlands, Poland, Portugal, Spain, and the United Kingdom	80	Mean age 38 years; 94% (75/80) women, 6% (5/80) men	ConnectEpeople (e-forum), Facebook, YouTube, Twitter, WhatsApp, Snapchat, and Instagram	Parents of children with illness	To identify the research priorities of parents of children with Down syndrome, cleft lip or cleft palate, congenital heart defects, or spina bifida	Approximately 2 months	105 parents were invited to secret Facebook group	92% (74/80) of participants accessed the survey through social media and Facebook members (32)	54 (68%) respondents (51.4% response rate)	Top 10 list of research priorities were successfully identified
Sylvia et al [32]	2018; United States	4103	Age range between 18 and 86 years; 78.21% (3209/4103) women, 19.01% (780/4103) men	Website and web-based forums	Patients, caregivers, clinicians, and other advocates	To understand research topics that are of most interest to individuals with mood disorders	May 2015 to May 2017 (24 months)	Not stated	4103 (100%) users enrolled into the web-based community (via the website)	Not stated	Research priority agenda in the area of mood disorders were successfully identified
Wojcieszek et al [33]	2019; Australia, New Zealand, Africa, Asia, Europe, North America, South or Central America, the United Kingdom, and Ireland	79	Not stated	Emails with embedded link	Individuals involved in stillbirth research, clinical practice, and advocacy	To identify research priorities and explore potential methodologies to inform care in subsequent pregnancies following a stillbirth	June 2018 to August 2018 (1.5 months)	124 email invitations were sent	Not stated	79 (100%) respondents (64% survey response rate)	Five priority research topics were successfully identified

^a^JM: juvenile myositis.

^b^JA: juvenile arthritis.

^c^cSLE: childhood-onset systemic lupus erythematosus.

^d^AF: Arthritis Foundation.

^e^LFA: Lupus Foundation of America.

^f^OGAA: oesophago-gastric anastomosis audit.

^g^RCT: randomized controlled trial.

^h^CF: cystic fibrosis.

^i^N/A: not applicable.

### Research Question 1: Social Media–Based Strategies Used

Table 2 shows the particular social media strategies used to enhance knowledge user engagement in research priority-setting exercises grouped by platform. Of studies using email as their primary social media platform [12,16,17,19,23,24,27,29,30,33] study teams emailed messages with embedded research prioritization survey links (including to researchers’ existing mailing lists) and integrated tell a friend tool in emails to prompt recipients to invite colleagues to participate. Facebook-specific methods to engage stakeholders included embedding survey links within Facebook posts, using the platform’s boosting feature (ie, paid advertisements), and hiring a Facebook advertising specialist. Informational Facebook pages were also used and involved private and public question-and-answer pages and a resource center with links to relevant documents [5,17,14-18].

Twitter-specific methods to engage participation included the use of hashtags within tweets and question-and-answer threads for prospective participants [5,14,15,17-19,25,31]. In addition, Salmi et al [27], hosted live chats on Twitter, in which host Twitter accounts tweet about predefined topics with questions during a set period, to which Twitter users respond via tweets and engage in discussions with each other. A web-based forum strategy led to the creation of a space where families and researchers could share ideas on the priority-setting research project [31]. Informational videos were created and hosted on YouTube for people potentially interested in contributing research priorities and were later posted on other platforms [28,29]. For studies involving blogs, researchers posted stories and internal updates related to the project to enhance interest in participation [28,29]. Studies also distributed e-newsletters to existing networks, sending them monthly to promote participation [16-18,28,30]. In addition, several studies used posts on Reddit and websites and web-based connection with the research team through video-calling platforms (eg, Skype, WhatsApp, or FaceTime or video chat on Facebook Messenger) to promote participation in priority-setting research [22,28,32].

Table 3 summarizes techniques to disseminate actual web-based research priority-setting surveys through social media. Snowball recruitment, in which current participants’ friends and family were approached for participation, was used [14,15,29,30]. Study teams also provided partner organizations with toolkits, templates, and promotional materials [5,12,13,15,17,23,29]. Then, organizations could use these materials to support the broadcasting of participation opportunities through social media. Individuals embedded in research prioritization exercises, such as steering group members, were additionally asked to promote the participation opportunity to their networks via social media [12-16,19,23,30], including by providing such individuals with preworded statements to tweet [19].

**Table 2:** Social media platform strategies.

Social media platform and specific strategy		Strategy description	Representative quotes	Studies providing evidence		
**Blogs**						
	Blog post stories	Posting insightful stories related to the priority-setting research project with the goal of promoting participation	“Weekly blogs by the chief executive officer profiling stories that are particularly moving or insightful, as well as internal news on the project.”	Shields et al [29]		
	Project news posting	Posting internal news or updates related to the priority-setting research project	“Some organisations or individuals promoted the study on Twitter or a blog.”	Dyson et al [14]		
**Emails**						
	Embedded links	Embedding survey links within emails to promote participation in the priority-setting research project	“Invitations to participate in the research and a link to the online survey (in the relevant language) were sent via email. Those approached to complete the survey were identified using membership lists of the African Palliative Care Association (APCA).”	Allsop et al [12], Correll et al [13]; Han et al [18], Kriss et al [21], Siefried et al [30], and Wojcieszek et al [33]		
	Mailing list distribution	The use of an existing mailing list to promote participation in the priority-setting research project	“A link to an initial electronic survey (created using REDCap) was emailed to members of Cure JM^a^, AF^b^ and LFA^c^ patient and family members and posted on their respective social media sites. The ranking survey was emailed to the Cure JM, AF, and LFA listservs and a link was posted on their respective social media sites.”	Allsop et al [12], Correll et al [13], Han et al [17], Siefried et al [30], and Wojcieszek et al [33]		
	Peer-to-peer dissemination	Using a *tell a friend tool*, which invites friends and colleagues to participate (peer-to-peer messaging) in the priority-setting research project	“Tell a Friend tool to invite friends or colleagues to participate, using e-mail-based peer-to-peer messaging.”	Shields et al [29]		
	Reminders to participate	Sending email reminders to individuals about the opportunity to participate in the priority-setting research project	“We sent an initial e-mail on Tuesday, January 30, 2017, at 12:00 PM EST to potential participants and, on subsequent Tuesdays between 10:00 AM and 12:00 PM EST, sent 5 weekly reminders to those who had not yet responded.”	Eberman et al [16], Han et al [17], Kriss et al [21], and Wojcieszek et al [33]		
	Reminders to finish survey	Sending email reminders to individuals who began the survey but only partially completed it	“Reminder emails were sent to non-responders and to individuals who began the survey but only partially completed it.”	Kriss et al [21] and Wojcieszek et al [33]		
**Facebook**						
	Embedded links to create ease of participation	Embedding simple and direct links within Facebook posts to external sites related to participation in the priority-setting research project	“Simple ‘How to Participate’ area that provided a visual menu of the ways to get involved, with simple links to take participants directly to the tools. Resource Centre page with access to links, documents and reports to help participants deepen their knowledge of the technical health challenges in the region.”	Normansell et al [5] and Shields et al [29]		
	Engagement of advertising strategists	Hiring a Facebook advertising strategist to plan the social media campaign used for promoting participation in the priority-setting research project	“Tactica Interactive, a digital media enterprise, was hired to broaden our sampling frame via a Facebook advertising strategy.”	Dyson et al [15]		
	Providing participation explanation	Creating a Facebook section that explains how to participate in the priority-setting research project	“Simple ‘How to Participate’ area that provided a visual menu of the ways to get involved, with simple links to take participants directly to the tools.”	Dyson et al [15]		
	Use of private and public pages	Creating both public and private Facebook groups to allow private discussion among participants in the priority-setting research project	“Announcement of the vEDS^d^ Collaborative survey was disseminated via vEDS public and private social media pages.” “Secret Facebook groups, providing optimal security, were set up for newly recruited research-aware parents (RAPs) to communicate privately and confidentially with each other and for the research team to generate questions and to interpret findings.”	Dyson et al, [14], Shalhub et al [28], and Sinclair et al [31]		
	Providing project explanation	Creating a section on Facebook page dedicated to explaining the priority-setting research project and how participation could have an impact	“‘About our Project’ section to provide participants with specific details on how their participation would affect the North West LHIN^e^ decision-making and the second IHSP^f^.”	Shields et al [29]		
	Question and answer	Using and moderating a web-based question-and-answer thread on Facebook to promote discussion topics regarding research participation	“To encourage engagement and re-engagement, the site moderator used online question and answer threads to keep promoting new discussion topics and emailed a weekly topic to all the registered users to encourage them to come back.”	Han et al [17] and Sinclair et al [31]		
	Resource center	Creating a resource center with links to documents and reports on the Facebook page	“‘Resource Centre’ page with access to links, documents and reports to help participants deepen their knowledge of the technical health challenges in the region.”	Shields et al [29]		
	Private and secret groups	Creating private Facebook groups to allow private discussion among participants in the priority-setting research project	“Announcement of the vEDS Collaborative survey was disseminated via vEDS public and private social media pages”	Shalhub et al [28] and Sinclair et al [31]		
**Newsletter**						
	Distribution through the researcher’s existing network	Distributing newsletter to an existing network to promote participation in the priority-setting research project	“To increase our reach and the likelihood of participation, the NATA^g^ marketing team distributed our recruitment announcement and link to volunteers via the ‘‘Range of Motion’’ newsletter to all registered attendees 5 and 6 weeks before the conference.”	Han et al [18], Eberman et al [16], and Siefried et al [30]		
	Frequent promotion	Sending monthly newsletters to promote participation in the priority-setting research project	“Social media promotion through Facebook and Twitter and monthly electronic newsletters from DiabetesSisters.”	Han et al [18] and Han et al [17]		
Web-based forums	Idea sharing	Creating forums through which families and researchers could share their ideas related to the priority-setting research project	“Moderated online group where families and researchers can share ideas related to research.”	Russell et al [26]		
Reddit	Posting of promotional material	The use of Reddit as a social media platform used to promote participation in the priority-setting research project	“Announcement of the vEDS Collaborative survey was disseminated via vEDS public and private social media pages.”	Shalhub et al [28]		
**Twitter**						
	Hashtags	Using Twitter hashtags to attract participants and generate conversation among relevant stakeholders	“A bespoke Twitter account was set up @questionCF with the associated hashtag #questionCF. This was managed by members of the steering group and aimed to promote the online surveys and increase participation.”	Rowbotham et al [25]		
	Question and answer	Creating a post for inviting participants to ask questions about the priority-setting research project, which was moderated by steering group members	“A bespoke Twitter account was set up @questionCF with the associated hashtag #questionCF. This was managed by members of the steering group and aimed to promote the online surveys and increase participation.”	Rowbotham et al [25]		
	Live chats	Host Twitter accounts tweeting about predefined topics with questions over a set period, during a scheduled chat, to which Twitter users respond via tweets and engage in discussions with each other. Tweets from participants are limited to 280 characters and participants typically include an assigned hashtag in their tweet, thus allowing aggregation of the conversation.	“The tweet chat hosts (@BTSMchat and @HPMchat, respectively) tweeted the 4 predefined topics (Table 1) with questions over a 60-minute period during a scheduled chat. The hosts alerted tweet chat participants that the transcript of the chat would be subject to qualitative analysis and used to inform research. One tweet question was posted roughly every 15 minutes. Twitter users responded to the questions and engaged in discussions with each other. On Twitter, responses are limited to 280 characters, and participants were instructed to add the #BTSM or #HPM hashtag to aggregate the conversation.”	Salmi et al [27]		
YouTube	Welcome video	Using YouTube to create a personal welcome message on Facebook pages, inviting users to participate in the priority-setting research project	“On the site’s home page, YouTube video personal welcome message.”	Shields et al [29] and Shalhub et al [28]		
Website	Posting of promotional material	Discussing the use of websites with survey as a social media platform used to promote participation in the priority-setting research project	“We created an online and social media presence via a study website (Outcomes in Child Health)...” “We collaborated with organisations interested in ARI^h^ and patient engagement to advertise our research via websites and other channels...”	Allsop et al [12], Dyson et al, Normansell et al [5], and Sylvia et al [32]		
Video calling	Digital connection to promote participation	Discussing the use of video-calling or internet-based face-to-face interactions to promote participation in the priority-setting research project	“Discussed details about the project and the parents’ research needs through face-to-face social media platforms such as Skype, WhatsApp, FaceTime, or via video chat on Facebook Messenger to build trust.”	Sinclair et al [31]		

^a^JM: juvenile myositis.

^b^AF: Arthritis Foundation.

^c^LFA: Lupus Foundation of America.

^d^vEDS: vascular Ehlers-Danlos syndrome.

^e^LHIN: local health integration network.

^f^IHSP: integrated health services plan.

^g^NATA: National Athletic Trainers’ Association.

^h^ARI: acute respiratory infection.

**Table 3:** Dissemination techniques.

Category and specific technique			Technique description		Representative quotes		Studies providing evidence
**Existing network**							
	Individual promotion	Using individuals (eg, steering group members) within existing network to promote the survey to their networks via social media		“Those approached to complete the survey were identified using membership lists of the African Palliative Care Association (APCA).” “A link to an initial electronic survey (created using REDCap) was emailed to members of Cure JM, AF and LFA patient and family listservs and posted on their respective social media sites.” “We also asked individuals and organisations within our existing networks to promote the study.” “All Steering Group members were requested to use pre-worded Tweets, which included the link to the survey.” “Invitations to participate in the research and a link to the online survey (in the relevant language) were sent via email. Those approached to complete the survey were identified using membership lists of the African Palliative Care Association (APCA).”		Allsop et al [12], Correll et al [13], Dyson et al, [14], Eberman et al [16], Healy et al [19], Rowbotham et al [25], and Siefried et al [30]	
	Individual promotion–prewording	Providing individuals (eg, steering group members) within existing network with preworded tweets to promote the research participation opportunity on their Twitter accounts		“All Steering Group members were requested to use pre-worded Tweets, which included the link to the survey.” “A bespoke Twitter account was set up @questionCF with the associated hashtag #questionCF. This was managed by members of the steering group and aimed to promote the online surveys and increase participation.”		Dyson et al [15]; Healy et al [19], Rowbotham et al [25], and Morse et al [23]	
**External organizations**							
	Social media collaboration	External organizations posting on their respective social media sites to promote research participation opportunity		“A link to an initial electronic survey (created using REDCap) was emailed to members of Cure JM, AF and LFA patient or family listservs and posted on their respective social media sites. The ranking survey was emailed to the Cure JM^a^, AF^b^, and LFA^c^ listservs and a link was posted on their respective social media sites.” “Tactica Interactive, a digital media enterprise, was hired to broaden our sampling frame via a Facebook advertising strategy.” “We collaborated with organisations interested in ARI^d^ and patient engagement to advertise our research via websites and other channels...” “A toolkit aimed at partnering organizations, which included a template for the invitation from the partner, a description of DiabetesSistersVoices, and promotional materials including flyers and postcards.” “A survey consisting of 27 questions was developed and distributed to surgeons from the OGAA^e^ collaborative and advertised through specialty organizations’ social media accounts”		Correll et al [13], Dyson et al [14], Han et al [17], Normansell et al [5], Siefried et al [30], and Oesophago-Gastric Anastomosis Study Group [24]	
	Providing resources	Providing external organizations with toolkits, templates, or promotional materials that serve as guidelines for when organization broadcasts research participation opportunity		“A toolkit aimed at partnering organizations, which included a template for the invitation from the partner, a description of DiabetesSistersVoices, and promotional materials including flyers and postcards.”		Han et al [17]	
	Website	External organizations posting on their website to promote research participation opportunity		“We collaborated with organisations interested in ARI and patient engagement to advertise our research via websites and other channels: The Alberta Centre for Child, Family & Community Research (now known as PolicyWise for Children and Families; a provincial organisation linking government, academia and the community in a focus on evidence-informed policy and practice),22 TRanslating Emergency Knowledge for Kids (a national network of researchers and clinicians invested in improving paediatric emergency care), 23 the Cochrane Consumer Network (an international network of healthcare consumers with an interest in evidence-based medicine) 24 and the Stollery Family Centered Care Network (a local children’s hospital-based network of patients and families that provide input into patient care).” “Online survey was posted on Survey Monkey and advertised through the Asthma UK Facebook and Twitter profiles and Cochrane Airways social media and website.”		Allsop et al [12], Dyson et al [14], and Normansell et al [5]	
Snowball recruitment	N/A^f^	Disseminating research opportunity to participants’ social networks to increase participation and access to specific populations		“We used snowball sampling to recruit parents.” “First, we focused on identifying and engaging recruitment targets with the potential for a high yield of participants. We then expanded our scope through referrals and diffusion via social media.” “Through Facebook, friend networks were encouraged to invite each other to participate.” “Tell a Friend tool to invite friends or colleagues to participate, using e-mail-based peer-to-peer messaging.”		Dyson et al [14], Shields et al [29], and Siefried et al [30]	
Boosts	N/A	Using the Facebook *boosting* feature to reach a wider audience of possible participants		“Facebook posts were “boosted” monthly to showcase the posts to more users.” “Social media promotion through Facebook and Twitter and monthly e-newsletters from DiabetesSisters Facebook posts were boosted to showcase the posts to more users, centralizing it to female users in the United States with interests in diabetes-relevant topics. DiabetesSisters posted on Facebook about the study and each month they “boosted” the post to increase the number of women who saw each post.”		Han et al [17] and Han et al [18]	

^a^JM: juvenile myositis.

^b^AF: Arthitis Foundation.

^c^LFA: Lupus Foundation of America.

^d^ARI: acute respiratory infection.

^e^OGAA: oesophago-gastric anastomosis audit.

^f^N/A: not applicable.

### Research Question 2: Measurement of Social Media Campaign Effectiveness

Across all the 23 included studies, 21 (91%) claimed to be successful in conducting health research priority-setting exercises via social media–based methods.

The direct effect of social media campaigns in securing stakeholder participation in research priority-setting was assessed as the (1) number of survey responses [12,14,15,20,33], (2) number of survey responses within a set period [14,15,20,33], (3) proportion of surveys fully completed [21], and (4) number of visits to external survey administration sites [14,15].

Indirect metrics for campaign effectiveness were (1) audience reach (ie, extent to which the survey sample was characteristic of the target population [13-15], number of countries and local communities represented in the sample [12,21], and number of national associations and external organizations contacted [12]); (2) campaign interaction (ie, number of clicks and impressions on posts [14,15,18,23,25,27], frequency of post views [26], volume of comments left by target stakeholders [26], number of searches for campaign pages or downloads of resources [17,18], number of bespoke hashtag clicks or uses [25,27], and Google Analytics [18]); (3) participant satisfaction [17,28,31]; and (4) platform-specific methods (ie, number of website views or likes [12,14,15,17-19,21,29], number of registered participants in an email chain or total number of delivered emails [12,13,16,19,21,29,33], new followers and likes on Facebook pages [14,15,17,18,26], and Twitter followers gained [14,15,25]).

### Research Question 3: Benefits, Limitations, and Recommendations

#### Benefits and Limitations of Social Media–Based Research Priority Setting

All included studies (23/23, 100%) successfully gathered research priorities from key stakeholders and knowledge users using social media–based participant recruitment. Cited benefits related to social media use were the capacity to elicit participation from many knowledge users [14,15,17,18,27,31], the speed at which research priorities were gathered, the sense of community developed [17,31], peer-support offered to patients and family members [17,26,28,31] by social media campaigns, and the capacity for dissemination of health-promoting resources from health care professionals to patients. A cited limitation of social media–based methods was that web-only methods may limit the participation of individuals with limited or no access to technology, limited leisure time to engage with social media, and lower socioeconomic status and of older age [12-15,17].

#### Recommendations for Successful Social Media–Based Research Priority Setting

To improve the effectiveness of social media campaigns, authors recommended focusing on the campaign’s graphic design components and style of messaging [26,31,32], creating opportunities for the target audience to personally interact with the team leading the campaign [31], and using platform-specific paid advertisements (ie, also termed *boosts*) [18,28].

Design-related recommendations included implementing illustrative and graphical sophistication, such as posts containing words, text, and video [31] and establishing a tone and style of graphics to create a consistent brand [26,32]. Messaging recommendations were to post some content that is not directly related to research, but of interest to community members—especially if these posts are community-led [22,26,31]; to avoid phrases that do not foster inclusivity and may separate the researchers from the target audience (ie, *us* vs *them* semantics); and to minimize scientific jargon in posts. Interaction-related recommendations involved using moderators [17,26], especially community members to build the authenticity of the campaign [27]; initiating conversations with perspective participants to *break the ice*; using software that supports face-to-face interaction between researchers and the community [31]; allowing peer-to-peer sharing (ie, providing community members with capacity to invite colleagues to participate) [17,22,26,28,29,31,33]; and using platform-specific boosts (eg, Facebook boosts) [18,28]. This last strategy corresponded with the highest recruitment and enrollment yields.

Recommendations to address the limitation that social media may prevent priority-setting participation by some groups were also suggested. These included implementing a hybrid of electronic and nonelectronic survey dissemination methods to increase the representation of those without access to technology [12,17,18], developing web-based materials with simple navigation requirements to allow participation by individuals with less experience with the web [30], and intentionally tailoring social media strategies (eg, hashtags and boosts) for subpopulations of individuals whom study teams identify as being underrepresented in research prioritization project data sets [13-15,17,21,25,32].

## Discussion

### Principal Findings

Recognizing the importance of engaging key stakeholders in developing research agendas, we sought to use the extant literature to understand how social media might support research priority-setting, how effectiveness of the method might be measured, and the method’s benefits and drawbacks. We show that multiple social media strategies, which differ depending on the social media platform, have been used to promote participation in research priority setting—with strong success rates in generating research agendas. Metrics to quantify the reach of these strategies included the number of impressions on posts (eg, likes and other reactions) and the volume of comments left by stakeholders. In addition to the benefits, limitations of the use of social media in research priority-setting were also identified. Results from this review can guide methods for research priority-setting by patients, family caregivers, health care professionals, and other advocates and support the engagement of these stakeholders in developing future research agendas.

### Social Media Platform Strategies and Dissemination Techniques

Social media–based strategies that incorporated platform-specific amplification (eg, Facebook boosts) and components that encouraged active engagement by participants (eg, question-and-answer forums and shared resources) enabled researchers to reach a broad audience of possible participants. This finding agrees with the literature showing that Facebook [34] health promotion posts receiving a paid boost reached significantly more users. Hashtags were also used in the included studies to increase visibility of tweets, which aligns with previous research showing hashtag use as effective in influencing social media conversations related to mental health [35] and in cases where the desired participant pool is small [36].

Our finding that snowball sampling is used to disseminate priority-setting surveys and expand participant pools aligns with other research showing that options to like, tag, or share posts expand a social media campaign’s reach [37]. This method may be particularly advantageous in cases where the campaign target audience is a specific and relatively small group (eg, people with lived experiences of less common diseases) and campaign participants may have contacts within their social network who they can engage in the process. Our results also suggest that there are priority-setting advantages in asking relevant external organizations and internal research and clinical team members to circulate survey links and use their personal or organization-affiliated social media accounts to expand reach.

### Measurement of Social Media Campaign Effectiveness

We identified several metrics used by researchers to evaluate the effectiveness of social media campaigns, including the number of post impressions, frequency of viewed posts, volume of comments left by stakeholders, and number of times a bespoke hashtag was clicked or used. The heterogeneity in metrics likely reflects the exponentially growing number of social media platforms. However, the collection and interpretation of these social media impact metrics support ongoing consideration of the campaign’s effectiveness and subsequent content adjustments to maximize campaign reach and engagement [35].

### Benefits and Limitations of and Recommendations for Social Media Campaigns in Research

Commonly identified benefits of priority-setting via social media include the speed at which participation opportunities can be disseminated and the capacity to build a sense of community among participants—possibly enhancing engagement. Research has also indicated that social media may be particularly useful in targeting information at some rarely reached groups such as individuals with depression [38]. In addition, moderators might humanize the campaign, build possible participant’s trust, and enhance campaign engagement by these individuals [39].

In contrast, limitations of social media–based methods for priority-setting research include the uncertainty of who is being captured through the posts [40]. Our study found that researchers commonly cite fears that social media–based methods may unexpectedly include or exclude the research priority perspectives of certain groups. In these cases, there are limited ways to assure that the recruited team of participants is the valid group of people that will render reliable results. This is problematic from ethical and methodological (ie, sampling bias) points of view and its mitigation requires careful planning. Moreover, when survey links are disseminated via social media, the true number of individuals that are reached cannot be calculated. This is because not all users will engage (ie, like, comment, and share) with the post [13,20]. In addition, although the platform analytics (ie, number of follows, comments, and likes on posts) are often used as an indication of survey engagement, these data may not be representative of the sample that opens the survey link or completes the survey.

Recommendations were also made to establish a consistent tone and branding, with a focus on using attractive graphic designs within priority-setting research campaigns. This consistency may increase the recognizability of the research project and authenticity to the effort, resulting in increased participation in priority-setting research efforts [41].

### Limitations of Our Study

The definition of social media varies substantially in the literature and some definitions used did not meet our inclusion criteria. Our conclusions regarding the recruitment for priority-setting research projects may differ from those arising if a different definition was used. Varying definitions of *social media* may also have rendered our decision-making process during the screening phase susceptible to error. However, we screened in duplicate with good consistency and used third-party arbitration of discrepancies. Finally, amid the COVID-19 pandemic, the number of studies adapting to web-based research methodologies, especially using social media, may have increased after the search strategy was performed. Considering such rapid growth, it is important to note that this review is a snapshot at a particular point in time that does not account for novel methods that may have emerged after our search.

### Recommendations for Practice and Future Research

Social media appears to be an effective means to recruit and involve participants in the research process. Thus, researchers should consider using web-based social networking as a method to recruit knowledge users, collect data, and translate knowledge into practice. The study team’s efforts to build knowledge user trust in prioritization efforts, including by humanizing the campaign through moderating chats and engaging with participants, may improve engagement. On the basis of our findings, efforts can be supported by optimizing the visual representation of data through illustrative posts containing text and graphics. Moreover, to enhance participation by a wide group of knowledge users, researchers should focus on developing accessible and inclusive web-based materials. In addition, investing in platform-specific boosts (eg, Facebook boosts) and paid advertisements may be an effective tactic to enhance participant recruitment and enrollment.

Given the relatively recent emergence of digital platforms, social media–based methods are understudied compared with traditional recruitment means. We have identified some possible limitations of the method, such as potential limited access to individuals of lower socioeconomic status or older age. However, few studies have determined the extent to which these limitations impact prioritization efforts and, in the case of older adults, contrary evidence exists indicating good engagement with social media and technologies [42]. Should the identified limitations of social media–based priority-setting be significant, research into ways to mitigate these shortcomings is needed. Further research is needed to understand how to enhance the capacity of social media recruitment to capture representative samples. More research is also needed to understand which social media strategies and dissemination techniques are likely to be successful for research prioritization efforts, with the understanding that these strategies and techniques are likely to change over time as new social media platforms and features become available. Finally, given the highly public nature of information exchange on social media, considerations of the data privacy and security implications of social media–based research prioritization efforts are needed.

### Conclusions

In this review, we synthesized the rapidly emerging data assessing the effectiveness of social media strategies to engage knowledge users in research priority-setting efforts across several social media platforms. The benefits of social media–based recruitment included the speed at which participation opportunities can be disseminated and the sense of community built among participants. As it is likely that social media–based research methods, including for research priority-setting, will be increasingly used by the scientific community, lessons and recommendations from this review can support scientists to more fully engage those who are most impacted by health research in setting associated research agendas.

## Appendix

Multimedia Appendix 1 Search strategy.

## Abbreviations

PRISMA-ScR: Preferred Reporting Items for Systematic Reviews and Meta-Analyses extension for Scoping Reviews
