# Supplementary material for: Using Social Media to Engage Knowledge Users in Health Research Priority Setting: Scoping Review
Source: J Med Internet Res. 2022 Feb 21;24(2):e29821. doi: 10.2196/29821 (PMC8902657; doi:10.2196/29821)
Supplement: Multimedia Appendix 1 [file jmir_v24i2e29821_app1.docx]

**Multimedia Appendix 1: Search Strategies**

**Embase**

**No.**

**Query**

**Results**

**682**

**#9**

**#1** AND **#5** AND **#8**

**785,317**

**#8**

**#6** OR **#7**

**587,177**

**#7**

**'patient recruitment'**/exp OR **recruit***:ti,ab,kw OR **'selection criteri*'**:ti,ab,kw

**215,093**

**#6**

((**patient*** OR **subject*** OR **community** OR **volunteer*** OR **stakeholder***) NEAR/3 (**engage*** OR **strateg*** OR **participat*** OR **selection**)):ti,ab,kw

**1,763,212**

**#5**

**#2** OR **#3** OR **#4**

**41,738**

**#4**

**facebook**:ti,ab,kw OR **google**:ti,ab,kw OR **youtube**:ti,ab,kw OR **whatsapp**:ti,ab,kw OR **tumblr**:ti,ab,kw OR **twitter**:ti,ab,kw OR **linkedin**:ti,ab,kw OR **instagram**:ti,ab,kw OR **pinterest**:ti,ab,kw OR **snapchat**:ti,ab,kw OR **reddit**:ti,ab,kw OR **qzone**:ti,ab,kw OR **weibo**:ti,ab,kw OR **wechat**:ti,ab,kw OR **youku**:ti,ab,kw OR **tudou**:ti,ab,kw OR **renren**:ti,ab,kw OR **badoo**:ti,ab,kw OR **baidu**:ti,ab,kw OR **orkut**:ti,ab,kw OR **foursquare**:ti,ab,kw OR **vine**:ti,ab,kw OR **vkontakte**:ti,ab,kw OR **myspace**:ti,ab,kw OR **bebo**:ti,ab,kw OR **hi5**:ti,ab,kw OR **tiktok**:ti,ab,kw OR **'tik tok'**:ti,ab,kw

**1,395,459**

**#3**

**'mass media'**:ti,ab,kw OR **'communicat* media'**:ti,ab,kw OR **telecommunicat***:ti,ab,kw OR **communicat***:ti,ab,kw OR **'social media'**:ti,ab,kw OR **'social network*'**:ti,ab,kw OR **mail***:ti,ab,kw OR **internet**:ti,ab,kw OR **web**:ti,ab,kw OR **telephon***:ti,ab,kw OR **cellphone***:ti,ab,kw OR **phone***:ti,ab,kw OR **smartphone***:ti,ab,kw OR **blog**:ti,ab,kw OR **blogs**:ti,ab,kw OR **blogging**:ti,ab,kw OR **television***:ti,ab,kw OR **tv**:ti,ab,kw OR **tvs**:ti,ab,kw OR **radio**:ti,ab,kw OR **video***:ti,ab,kw OR **messag***:ti,ab,kw OR **announce***:ti,ab,kw OR **ad**:ti,ab,kw OR **ads**:ti,ab,kw OR **advertis***:ti,ab,kw OR **campaign***:ti,ab,kw OR **texting**:ti,ab,kw OR **videoconferenc***:ti,ab,kw OR **webcast***:ti,ab,kw OR **news**:ti,ab,kw OR **newsletter***:ti,ab,kw OR (((**smartphone*** OR **phone*** OR **mobile**) NEAR/2 (**app** OR **apps** OR **application***)):ti,ab,kw)

**568,972**

**#2**

**'mass communication'**/exp

**22,336**

**#1**

**'priority setting'**/exp OR (((**priorit*** OR **research**) NEXT/2 (**set** OR **setting** OR **partnership*** OR **agenda***)):ti,ab,kw) OR ((**research** NEAR/2 **priorit***):ti,ab,kw)

**PubMed**

| Search | Query | Results |
| --- | --- | --- |
| #3 | Search: **#1 AND #2 AND #3** | [688](https://pubmed.ncbi.nlm.nih.gov/?term=%2316+AND+%2317+AND+%2323&sort=) |
| #2 | Search: **"priority research"[tiab] or "priorities research"[tiab] or "research priority"[tiab] or "research priorities"[tiab] or "priority set"[tiab] or "priority setting"[tiab] or "priority partnership"[tiab] or "priority partnerships"[tiab] or "priority agenda"[tiab] or "priority agendas"[tiab] or "priorities set"[tiab] or "priorities setting"[tiab] or "priorities partnerships"[tiab] or "priorities partnership"[tiab] or "priorities agenda"[tiab] or "priorities agendas"[tiab] or "research set"[tiab] or "research setting"[tiab] or "research partnership"[tiab] or "research partnerships"[tiab] or "research agenda"[tiab] or "research agendas"[tiab]** | [16,191](https://pubmed.ncbi.nlm.nih.gov/?term=%22priority+research%22%5Btiab%5D+or+%22priorities+research%22%5Btiab%5D+or+%22research+priority%22%5Btiab%5D+or+%22research+priorities%22%5Btiab%5D+or+%22priority+set%22%5Btiab%5D+or+%22priority+setting%22%5Btiab%5D+or+%22priority+partnership%22%5Btiab%5D+or+%22priority+partnerships%22%5Btiab%5D+or+%22priority+agenda%22%5Btiab%5D+or+%22priority+agendas%22%5Btiab%5D+or+%22priorities+set%22%5Btiab%5D+or+%22priorities+setting%22%5Btiab%5D+or+%22priorities+partnerships%22%5Btiab%5D+or+%22priorities+partnership%22%5Btiab%5D+or+%22priorities+agenda%22%5Btiab%5D+or+%22priorities+agendas%22%5Btiab%5D+or+%22research+set%22%5Btiab%5D+or+%22research+setting%22%5Btiab%5D+or+%22research+partnership%22%5Btiab%5D+or+%22research+partnerships%22%5Btiab%5D+or+%22research+agenda%22%5Btiab%5D+or+%22research+agendas%22%5Btiab%5D&sort=) |
| #1 | Search: **(((patient*[tiab] OR subject*[tiab] OR community[tiab] OR volunteer*[tiab] OR stakeholder*[tiab]) AND (engage*[tiab] OR strateg*[tiab] OR participat*[tiab] OR selection*[tiab]))) OR ("patient selection"[mesh] or recruit*[tiab] OR 'selection criteria'[tiab] or 'selection criterium'[tiab])** | [1,240,174](https://pubmed.ncbi.nlm.nih.gov/?term=%28%28%28patient%2A%5Btiab%5D+OR+subject%2A%5Btiab%5D+OR+community+OR+volunteer%2A%5Btiab%5D+OR+stakeholder%2A%5Btiab%5D%29+AND+%28engage%2A%5Btiab%5D+OR+strateg%2A%5Btiab%5D+OR+participat%2A%5Btiab%5D+OR+selection%2A%5Btiab%5D%29%29%29+OR+%28%22patient+selection%22%5Bmesh%5D+or+recruit%2A%5Btiab%5D+OR+%27selection+criteria%27%5Btiab%5D+or+%27selection+criterium%27%5Btiab%5D%29&sort=) |
| #16 | Search: **((telecommunications[MeSH] or "social media"[MeSH] or "mass media"[MeSH] or "blogging"[MeSH] or "communications media"[MeSH:noexp]) OR ("mass media"[tiab] or "communication media"[tiab] or "communications media"[tiab] or telecommunicat*[tiab] OR communicat*[tiab] OR 'social media'[tiab] OR 'social networks'[tiab] OR 'social network'[tiab] OR mail*[tiab] OR internet[tiab] OR web[tiab] OR telephon*[tiab] OR cellphone*[tiab] OR phone*[tiab] OR smartphone*[tiab] OR blog[tiab] OR blogs[tiab] OR blogging[tiab] OR television*[tiab] OR tv[tiab] OR tvs[tiab] OR radio[tiab] OR video*[tiab] OR messag*[tiab] OR announce*[tiab] OR ad[tiab] OR ads[tiab] OR advertis*[tiab] OR campaign*[tiab] OR texting[tiab] OR videoconferenc*[tiab] OR webcast*[tiab] OR news[tiab] OR newsletter*[tiab] OR ((smartphone*[tiab] OR phone*[tiab] OR mobile[tiab]) AND (app[tiab] OR apps[tiab] OR application*[tiab])))) OR (facebook[tiab] OR google[tiab] OR youtube[tiab] OR whatsapp[tiab] OR tumblr[tiab] OR twitter[tiab] OR linkedin[tiab] OR instagram[tiab] OR pinterest[tiab] OR snapchat[tiab] OR reddit[tiab] OR qzone[tiab] OR weibo[tiab] OR wechat[tiab] OR youku[tiab] OR tudou[tiab] OR renren[tiab] OR badoo[tiab] OR baidu[tiab] OR orkut[tiab] OR foursquare[tiab] OR vine[tiab] OR vkontakte[tiab] OR myspace[tiab] OR bebo[tiab] OR hi5[tiab] OR tiktok[tiab] OR 'tik tok'[tiab])** | [1,106,312](https://pubmed.ncbi.nlm.nih.gov/?term=%28%28telecommunications%5BMeSH%5D+or+%22social+media%22%5BMeSH%5D+or+%22mass+media%22%5BMeSH%5D+or+%22blogging%22%5BMeSH%5D+or+%22communications+media%22%5BMeSH%3Anoexp%5D%29+OR+%28%22mass+media%22%5Btiab%5D+or+%22communication+media%22%5Btiab%5D+or+%22communications+media%22%5Btiab%5D+or+telecommunicat%2A%5Btiab%5D+OR+communicat%2A%5Btiab%5D+OR+%27social+media%27%5Btiab%5D+OR+%27social+networks%27%5Btiab%5D+OR+%27social+network%27%5Btiab%5D+OR+mail%2A%5Btiab%5D+OR+internet%5Btiab%5D+OR+web%5Btiab%5D+OR+telephon%2A%5Btiab%5D+OR+cellphone%2A%5Btiab%5D+OR+phone%2A%5Btiab%5D+OR+smartphone%2A%5Btiab%5D+OR+blog%5Btiab%5D+OR+blogs%5Btiab%5D+OR+blogging%5Btiab%5D+OR+television%2A%5Btiab%5D+OR+tv%5Btiab%5D+OR+tvs%5Btiab%5D+OR+radio%5Btiab%5D+OR+video%2A%5Btiab%5D+OR+messag%2A%5Btiab%5D+OR+announce%2A%5Btiab%5D+OR+ad%5Btiab%5D+OR+ads%5Btiab%5D+OR+advertis%2A%5Btiab%5D+OR+campaign%2A%5Btiab%5D+OR+texting%5Btiab%5D+OR+videoconferenc%2A%5Btiab%5D+OR+webcast%2A%5Btiab%5D+OR+news%5Btiab%5D+OR+newsletter%2A%5Btiab%5D+OR+%28%28smartphone%2A%5Btiab%5D+OR+phone%2A%5Btiab%5D+OR+mobile%5Btiab%5D%29+AND+%28app%5Btiab%5D+OR+apps%5Btiab%5D+OR+application%2A%5Btiab%5D%29%29%29%29+OR+%28facebook%5Btiab%5D+OR+google%5Btiab%5D+OR+youtube%5Btiab%5D+OR+whatsapp%5Btiab%5D+OR+tumblr%5Btiab%5D+OR+twitter%5Btiab%5D+OR+linkedin%5Btiab%5D+OR+instagram%5Btiab%5D+OR+pinterest%5Btiab%5D+OR+snapchat%5Btiab%5D+OR+reddit%5Btiab%5D+OR+qzone%5Btiab%5D+OR+weibo%5Btiab%5D+OR+wechat%5Btiab%5D+OR+youku%5Btiab%5D+OR+tudou%5Btiab%5D+OR+renren%5Btiab%5D+OR+badoo%5Btiab%5D+OR+baidu%5Btiab%5D+OR+orkut%5Btiab%5D+OR+foursquare%5Btiab%5D+OR+vine%5Btiab%5D+OR+vkontakte%5Btiab%5D+OR+myspace%5Btiab%5D+OR+bebo%5Btiab%5D+OR+hi5%5Btiab%5D+OR+tiktok%5Btiab%5D+OR+%27tik+tok%27%5Btiab%5D%29&sort=) |

**Cochrane**

#1 MeSH descriptor: [Communications Media] this term only

#2 MeSH descriptor: [Telecommunications] explode all trees

#3 MeSH descriptor: [Social Media] explode all trees

#4 MeSH descriptor: [Mass Media] explode all trees

#5 MeSH descriptor: [Blogging] explode all trees

#6 ("mass media" or "communication media" or "communications media" or telecommunicat* OR communicat* OR "social media" OR "social networks" OR "social network" OR mail* OR internet OR web OR telephon* OR cellphone* OR phone* OR smartphone* OR blog OR blogs OR blogging OR television* OR tv OR tvs OR radio OR video* OR messag* OR announce* OR ad OR ads OR advertis* OR campaign* OR texting OR videoconferenc* OR webcast* OR news OR newsletter* OR ((smartphone* OR phone* OR mobile) AND (app OR apps OR application*)) OR facebook OR google OR youtube OR whatsapp OR tumblr OR twitter OR linkedin OR instagram OR pinterest OR snapchat OR reddit OR qzone OR weibo OR wechat OR youku OR tudou OR renren OR badoo OR baidu OR orkut OR foursquare OR vine OR vkontakte OR myspace OR bebo OR hi5 OR tiktok OR "tik tok"):ti,ab,kw

#7 #1 OR #2 OR #3 OR #4 OR #5 OR #6

#8 MeSH descriptor: [Patient Selection] explode all trees

#9 ("selection criteri*" OR recruit* OR ((patient* OR subject* OR community OR volunteer* OR stakeholder*) AND (engage* OR strateg* OR participat* OR selection*))):ti,ab,kw

#10 #8 OR #9

#11 (((priorit* OR research) NEXT/2 (set OR setting OR partnership* OR agenda*))):ti,ab,kw OR ((research NEAR/2 priorit*)):ti,ab,kw

#12 #7 AND #10 AND #11

**Scopus**

( ( TITLE-ABS-KEY ( ( ( priorit*  OR  research )  PRE/2  ( set  OR  setting  OR  partnership*  OR  agenda* ) ) ) )  OR  ( TITLE-ABS-KEY ( research  W/2  priorit* ) ) )  AND  ( TITLE-ABS-KEY ( "mass media"  OR  "communication media"  OR  "communications media"  OR  telecommunicat*  OR  communicat*  OR  "social media"  OR  "social networks"  OR  "social network"  OR  mail*  OR  internet  OR  web  OR  telephon*  OR  cellphone*  OR  phone*  OR  smartphone*  OR  blog  OR  blogs  OR  blogging  OR  television*  OR  tv  OR  tvs  OR  radio  OR  video*  OR  messag*  OR  announce*  OR  ad  OR  ads  OR  advertis*  OR  campaign*  OR  texting  OR  videoconferenc*  OR  webcast*  OR  news  OR  newsletter*  OR  ( ( smartphone*  OR  phone*  OR  mobile )  W/2  ( app  OR  apps  OR  application* ) )  OR  facebook  OR  google  OR  youtube  OR  whatsapp  OR  tumblr  OR  twitter  OR  linkedin  OR  instagram  OR  pinterest  OR  snapchat  OR  reddit  OR  qzone  OR  weibo  OR  wechat  OR  youku  OR  tudou  OR  renren  OR  badoo  OR  baidu  OR  orkut  OR  foursquare  OR  vine  OR  vkontakte  OR  myspace  OR  bebo  OR  hi5  OR  tiktok  OR  "tik tok" ) )  AND  ( TITLE-ABS-KEY ( "selection criteri*"  OR  recruit*  OR  ( ( patient*  OR  subject*  OR  community  OR  volunteer*  OR  stakeholder* )  W/3  ( engage*  OR  strateg*  OR  participat*  OR  selection* ) ) ) )

**Web of Science**

| Set | Results |  |
| --- | --- | --- |

| # 6 | [789](http://apps.webofknowledge.com.laneproxy.stanford.edu/summary.do?product=WOS&doc=1&qid=12&SID=7AAXt1i5hoarUVibVlE&search_mode=CombineSearches&update_back2search_link_param=yes) | #5  AND  #4  AND  #3  Indexes=SCI-EXPANDED, SSCI, A&HCI, CPCI-S, CPCI-SSH, ESCI Timespan=All years |
| --- | --- | --- |
| # 5 | [670,024](http://apps.webofknowledge.com.laneproxy.stanford.edu/summary.do?product=WOS&doc=1&qid=11&SID=7AAXt1i5hoarUVibVlE&search_mode=AdvancedSearch&update_back2search_link_param=yes) | TS=("selection criteri*"  OR  recruit*  OR  ((patient* OR subject* OR community OR volunteer* OR stakeholder* )  NEAR/3  (engage* OR strateg* OR participat* OR selection*) ))  Indexes=SCI-EXPANDED, SSCI, A&HCI, CPCI-S, CPCI-SSH, ESCI Timespan=All years |
| # 4 | [3,317,742](http://apps.webofknowledge.com.laneproxy.stanford.edu/summary.do?product=WOS&doc=1&qid=8&SID=7AAXt1i5hoarUVibVlE&search_mode=AdvancedSearch&update_back2search_link_param=yes) | TS=("mass media"  OR  "communication  media"  OR  "communications  media"  OR  telecommunicat*  OR  communicat*  OR  "social  media"  OR  "social  networks"  OR  "social  network"  OR  mail*  OR  internet  OR  web  OR  telephon*  OR  cellphone*  OR  phone*  OR  smartphone*  OR  blog  OR  blogs  OR  blogging  OR  television*  OR  tv  OR  tvs  OR  radio  OR  video*  OR  messag*  OR  announce*  OR  ad  OR  ads  OR  advertis*  OR  campaign*  OR  texting  OR  videoconferenc*  OR  webcast*  OR  news  OR  newsletter*  OR  ((smartphone* OR phone* OR mobile)  NEAR/2  (app OR apps OR application*) )  OR  facebook  OR  google  OR  youtube  OR  whatsapp  OR  tumblr  OR  twitter  OR  linkedin  OR  instagram  OR  pinterest  OR  snapchat  OR  reddit  OR  qzone  OR  weibo  OR  wechat  OR  youku  OR  tudou  OR  renren  OR  badoo  OR  baidu  OR  orkut  OR  foursquare  OR  vine  OR  vkontakte  OR  myspace  OR  bebo  OR  hi5  OR  tiktok  OR  "tik  tok")  Indexes=SCI-EXPANDED, SSCI, A&HCI, CPCI-S, CPCI-SSH, ESCI Timespan=All years |
| # 3 | [70,611](http://apps.webofknowledge.com.laneproxy.stanford.edu/summary.do?product=WOS&doc=1&qid=7&SID=7AAXt1i5hoarUVibVlE&search_mode=CombineSearches&update_back2search_link_param=yes) | #2  OR  #1  Indexes=SCI-EXPANDED, SSCI, A&HCI, CPCI-S, CPCI-SSH, ESCI Timespan=All years |
| # 2 | [14,240](http://apps.webofknowledge.com.laneproxy.stanford.edu/summary.do?product=WOS&doc=1&qid=6&SID=7AAXt1i5hoarUVibVlE&search_mode=AdvancedSearch&update_back2search_link_param=yes) | TS=(research   NEAR/2  priorit*)  Indexes=SCI-EXPANDED, SSCI, A&HCI, CPCI-S, CPCI-SSH, ESCI Timespan=All years |
| # 1 | [58,481](http://apps.webofknowledge.com.laneproxy.stanford.edu/summary.do?product=WOS&doc=1&qid=5&SID=7AAXt1i5hoarUVibVlE&search_mode=AdvancedSearch&update_back2search_link_param=yes) | TS=(((priorit*   OR  research)  NEAR/2  (set OR setting OR partnership* OR agenda*) ))  Indexes=SCI-EXPANDED, SSCI, A&HCI, CPCI-S, CPCI-SSH, ESCI Timespan=All years |
